# Supplementary material for: Valproic Acid Causes Redox-Regulated Post-Translational Protein Modifications That Are Dependent upon P19 Cellular Differentiation States
Source: Antioxidants (Basel). 2024 May 1;13(5):560. doi: 10.3390/antiox13050560 (PMC11117966; doi:10.3390/antiox13050560)
Supplement: Supplementary file 1 [file antioxidants-13-00560-s001.zip › antioxidants-2971326-supplementary/antioxidants-2971326-supplementary.pdf]

antioxidants

MDPI

Supplementary Materials

Supplementary Methods

Cell Viability Assay

To assess D3T and VPA toxicity, undifferentiated P19 cells and P19-derived neurons were cultured in 96-well cell culture plates (Genesee Scientific) and exposed to increasing concentrations of D3T (5–200 µM) or VPA (2.5–50 mM) for 24 h. Select cells were treated with H_2O_2 (200 µM) for 24 h as a positive control. Following exposure, cell viability was measured using 3-(4,5-dimethylthiazol-2-yl)-2,5-diphenyltetrazolium bromide (MTT; Sigma-Aldrich) [71]. At the end of the treatment period, MTT (0.5 mg/mL) was added to the medium and incubated at 37 °C for 3 h. The culture medium was removed and the exposed formazan crystals were dissolved in 100 µL of DMSO. The absorbance of each well was measured at 570 nm using a Spectramax iD3 Microplate Reader (Molecular Devices, San Jose, CA, USA).

Intracellular ROS Assay

Undifferentiated and differentiated cells were cultured in 96-well cell culture plates to measure ROS generation using 2′,7′-dichlorofluorescein (DCF) fluorescence as described previously [72]. Select wells were pretreated with D3T (10 µM) or vehicle (DMSO) for 12 h prior to the start of the assay. The medium was changed to Alpha Minimum Essential Medium containing 1% FBS and cells were preloaded with DCF diacetate (5 µM; Sigma-Aldrich) for 20 min. Following the 20 min DCF diacetate preloading, cells were washed with PBS and then treated with varying concentrations of VPA (2.5–50 mM). Select cells were also treated with H_2O_2 (200 µM) as a positive control. Fluorescence was measured at 485 nm excitation and 530 nm emission using a Spectramax iD3 Microplate Reader (Molecular Devices) before and after the 20-min VPA treatment. The change in florescence was calculated between the 0- and 20-min readings for each sample.

NRF2 Luciferase Reporter Assay

The transduction of cell cultures was performed following the instructions provided by the lentivirus supplier (BPS Bioscience, San Diego, CA, USA). In brief, undifferentiated P19 cells were seeded at a concentration of 10000 cells per well of a 96-well cell culture plate and incubated overnight. The following day, ARE Luciferase Reporter Lentivirus (10 µL per well; BPS Bioscience) and polybrene (5 µg/mL; Sigma-Aldrich) were added to each well and incubated for 24 h. Cells were then washed with PBS and transferred to 60 mm cell culture dishes. To ensure lentiviral transduction, cells were grown in growth medium containing puromyocin (2 µg/mL; Sigma-Aldrich) for four days. Following antibiotic selection, transduced cells were differentiated into neurons. Undifferentiated P19 cells and P19-derived neurons were transferred to 96-well cell culture plates and treated with D3T (10 µM) or vehicle (DMSO) overnight. Following treatment, luciferase activity was determined via the ONE-Step Luciferase Assay System (BPS Biosciences). Luminescence was measured using a Spectramax iD3 Microplate Reader (Molecular Devices).

Quantitative Real-Time PCR

To assess NRF2 induction with D3T treatment, undifferentiated P19 cells and P19-derived neurons were treated with D3T (10 µM) or vehicle (DMSO) for 12 h, after which cells were collected in TRIzol Reagent. Sample RNA was extracted using Direct-zol RNA MiniPrep Kits and RNA concentrations were determined using a NanoDrop ND-1000 Spectrophotometer. The isolated RNA was synthesized into cDNA using iScript cDNA Synthesis Kits and the cDNA was then used for quantitative real-time PCR using SYBR Green Detection Master Mixes in a StepOnePlus Real-Time PCR System. All primers were synthesized by and purchased from Integrated DNA Technologies and used at a final

concentration of 10 μM (Table S1). The genes assessed are considered hallmark indicators of NRF2 activity [73–75]: glutamate-cysteine ligase catalytic subunit (*Gclc*), heme oxygenase 1 (*Hmox1*), and NAD(P)H dehydrogenase [quinone] 1 (*Nqo1*). Beta-actin was measured as a housekeeping gene for normalization purposes. Samples were analyzed using the $\Delta\Delta C_t$ method [76].

Immunoblotting

Undifferentiated and differentiated cells were treated with D3T (10 μM) or vehicle (DMSO) for 12 h, collected in RIPA lysis buffer containing protease inhibitors (Roche Diagnostics), briefly sonicated, and finally centrifuged at 10000 × *g* and 4 °C for 5 min. Protein concentrations in the collected supernatants were determined using BCA Protein Assay Kits. Equal amounts of protein were separated by polyacrylamide gel electrophoresis and transferred onto nitrocellulose membranes. Membranes were blocked in blocking buffer at 4 °C overnight. After blocking, membranes were incubated with antibodies against POU domain, class 5, transcription factor 1 (OCT4; Abcam #19857, Waltham, MA, USA), tubulin beta-3 chain (βIII-tubulin; Abcam #18207), GCLC (Abcam #190685), HMOX1 (Abcam #13248), NQO1 (Abcam #28947), and beta-actin (Santa Cruz Biotechnology #B0904, Dallas, TX, USA) at concentrations of 1:500 in PBST at room temperature for 1.5 h. Membranes were washed three consecutive times with PBST and then incubated with secondary antibodies: Alexa Fluor 680 donkey anti-goat (Abcam #175776), 800 donkey anti-mouse (Abcam #216774), or 800 goat anti-rabbit (Abcam #216773) at concentrations of 1:5,000 in PBST at room temperature for 1.5 h. Membranes were again washed three times with PBST and then imaged on an Odyssey CLx Infrared Imaging System and quantified by expression of the desired protein relative to the beta-actin loading control.

RNA-Sequencing

Treated cells were collected in TRIzol Reagent and RNA was extracted using Direct-zol RNA Miniprep Kits. RNA concentration and integrity were measured using a NanoDrop ND-1000 Spectrophotometer. All samples submitted for sequencing had an RNA integrity number > 9. Library construction and sequencing were performed by NovoGene. In brief, poly(A)-containing RNA was isolated from 500 ng of total RNA, fragmented, and synthesized into cDNA. Unique indexing adapters were ligated to the cDNA and the samples were expanded by PCR amplification. Each library was sequenced as paired-end, 150- base-pair reads using a NovaSeq 6000 System.

Differential Gene Expression Analysis and Clustering

Read quality was assessed using FastQC (v0.12) and the reads from each sample were mapped independently to the mouse reference transcriptome (Ensembl, Mus musculus version 103) using Kallisto (v0.48.0). The results of transcript quantification were summarized to genes using Tximeta (v1.16.1). Differential gene expression was evaluated using the Wald test through DESeq2 (v1.40.2). Genes with an adjusted *P* value < 0.05 were designated as differentially expressed. Clust (v1.18.0) was used to analyze co-regulated genes and enrichment analysis of select clusters was performed using ClusterProfiler (v4.8.2). Data were plotted using ggplot2 (v3.5.0).

Statistical Analyses

The R computational software environment (v4.3.3) was used for all statistical analyses. Homoscedasticity for each experiment was assessed using a Shaphiro-Wilk test. Statistical comparisons were made using either one-way ANOVA followed by pairwise t-tests using the Bonferroni correction, or two-way ANOVA followed by Tukey's HSD post hoc tests. Quantitative data are presented as means ± SEM. Asterisks denote a statistically significant difference (* = $p < 0.05$, ** = $p < 0.01$, and *** = $p < 0.001$).

Supplementary Table

Table S1. Forward and reverse primer sequences for select NRF2-regulated genes and beta-actin.

Gene names	Forward primer sequences (5′ to 3′)	Reverse primer sequences (5′ to 3′)
Gclc	GACTGTTGCCAGGTGGATGA	ACCCTCTCTCTCTCTGCTGG
Hmox1	GTTCAAGAGGAGCAGAAGAA-GAAC	TTCTAGCTTTGATCTGGTTGTCAG
Nqo1	AGCCCCACCAAGTTCAAACA	AAGTGACGCCATCTGTGAGG
Beta-actin	GACTACCTCATGAAGATCCTGACC	ACATAGCACAGCTTCTCTTT-GATG

Supplementary Figures

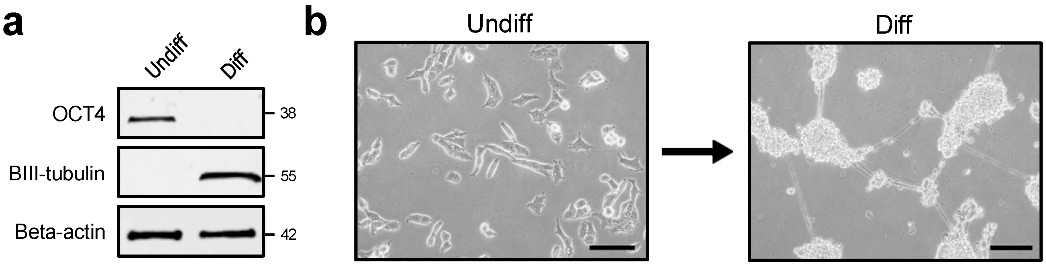

Figure S1. P19 cells successfully differentiate into neurons. (**a**) Immunoblotting depicting POU domain, class 5, transcription factor 1 (OCT4), a marker of cell stemness, and tubulin beta-3 chain (βIII-tubulin), a marker of neuronal differentiation, in undifferentiated P19 cells and P19-derived neurons ($n = 3$). (**b**) Morphological differences between undifferentiated cells on day 0 and neurons on day 6 of differentiation. Scale bars represent 50 µm. Images are representative of at least three independent experiments.

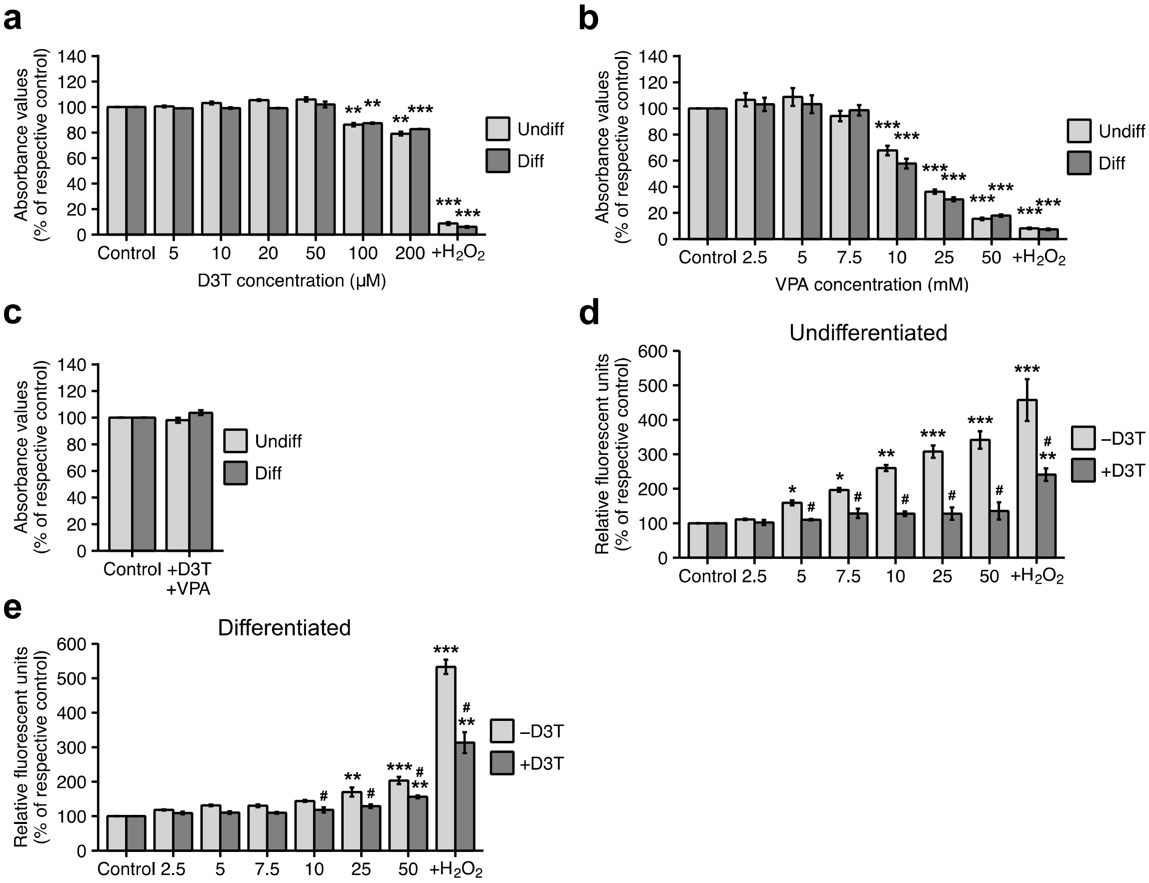

Figure S2. Valproic acid increases reactive oxygen species (ROS) generation in undifferentiated cells and differentiated neurons. (**a**,**b**) Undifferentiated P19 cells and P19-derived neurons were treated with varying concentrations of D3T (**a**) or VPA (**b**) for 24 h to assess cell viability ($n = 5$). (**c**) Cells

were co-treated with 10 μM D3T and 5 mM VPA, the concentrations used for all subsequent experiments, for 24 h but exhibited no statistical difference in viability compared to controls (n = 5). (**d**,**e**) Undifferentiated (**d**) and differentiated (**e**) cells were pretreated with D3T or vehicle and then treated with increasing concentrations of VPA to measure ROS generation over 20 min (n = 5). Exposure to H_2O_2 (200 μM) was used as a positive control (**a**,**b**,**d**,**e**). Data are presented as means ± SEM (**a**–**e**). Statistical comparisons were made using. a one-way ANOVA followed by a pairwise t-test using the Bonferroni correction (**a**–**c**) and a two-way ANOVA followed by a Tukey HSD post hoc test (**d**,**e**). Asterisks denote a statistically significant difference (* = $p < 0.05$, ** = $p < 0.01$, and *** = $p < 0.001$) compared to respective vehicle-treated controls, whereas number signs denote a statistically significant difference (# for $p < 0.05$) between combined D3T and VPA treatment compared to VPA alone of the same treatment concentration.

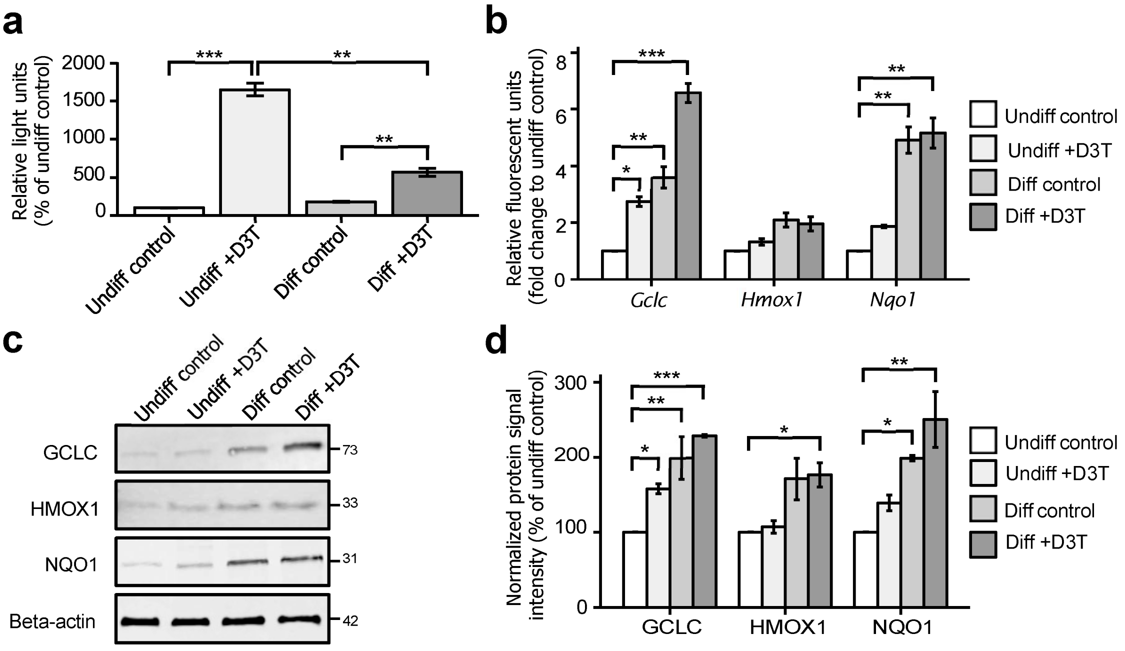

Figure S3. Treatment with D3T induces the NRF2 antioxidant pathway regardless of cellular differentiation state. (**a**–**d**) Undifferentiated cells and differentiated neurons were treated with D3T and assessed for NRF2 induction via luciferase activity (n = 3; (**a**)) and through gene (n = 3; (**b**)) and protein (n = 5; (**c**,**d**)) expression. Images are representative of at least three independent experiments (**c**). Data are presented as means ± SEM (**a**,**b**,**d**). Statistical comparisons were made using a one-way ANOVA followed by a pairwise t-test using the Bonferroni correction (**a**,**b**,**d**). Asterisks denote a statistically significant difference (* = $p < 0.05$, ** = $p < 0.01$, and *** = $p < 0.001$).

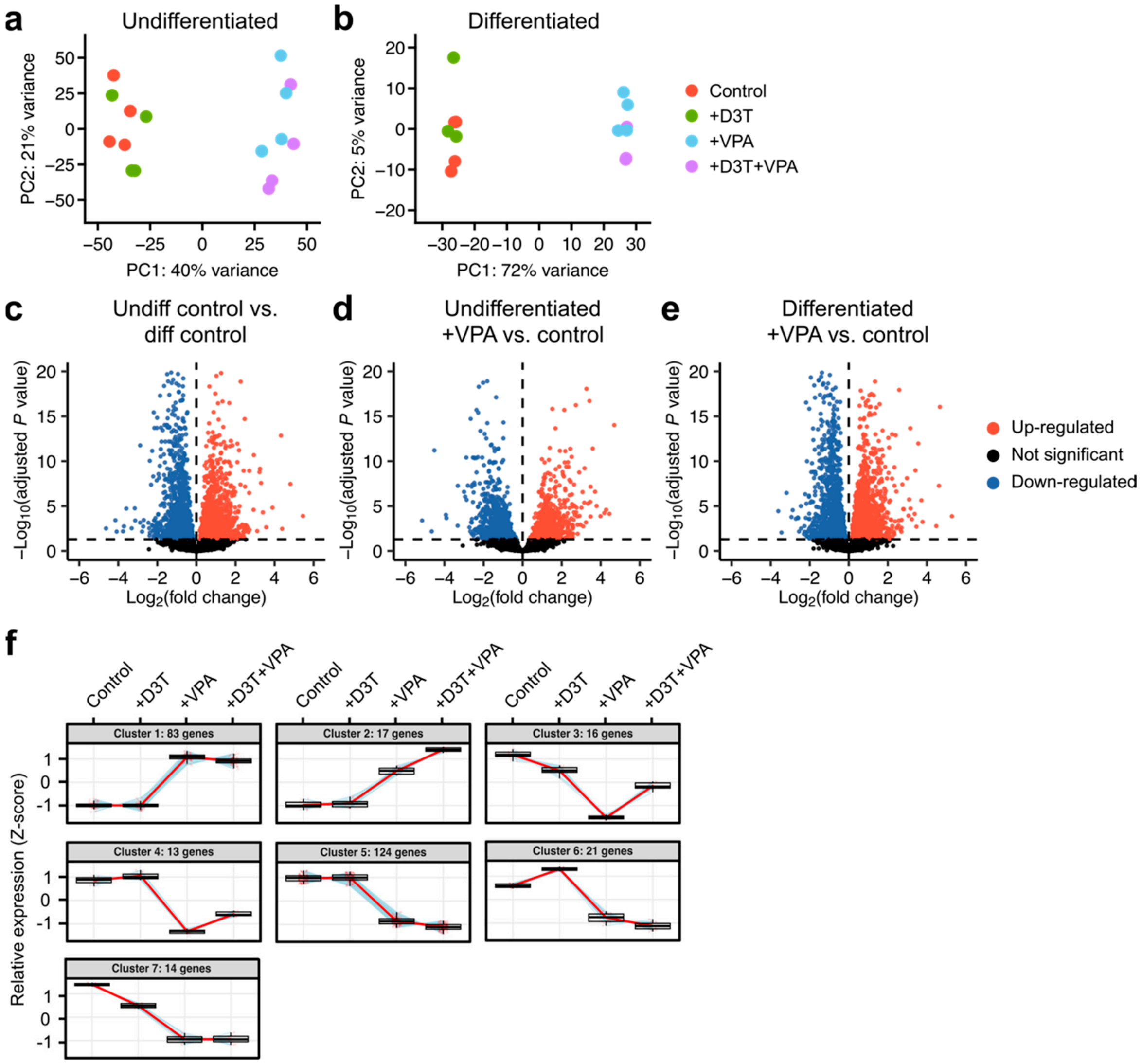

Figure S4. Valproic acid causes transcriptional alterations in both undifferentiated and differentiated cells. Undifferentiated cells and differentiated neurons were treated and analyzed using bulk RNA-sequencing. (**a**,**b**) Undifferentiated (n = 4; (**a**)) and differentiated (n = 3–4; (**b**)) sample-to-sample variance was visualized using principal component analysis plots. (**c**) Undifferentiated control samples are transcriptionally different compared to their differentiated counterparts. (**d**,**e**) Treatment with VPA causes differential gene expression in both undifferentiated (**d**) and differentiated samples (**e**). (**f**) Differentiated sample gene clustering reveals no groups that are significantly protected from VPA exposure by D3T pretreatment.
